# Large Scale Association Analysis for Drug Addiction: Results from SNP to Gene

**DOI:** 10.1100/2012/939584

**Published:** 2012-12-27

**Authors:** Xiaobo Guo, Zhifa Liu, Xueqin Wang, Heping Zhang

**Affiliations:** ^1^Department of Biostatistics, Yale University School of Public Health, New Haven, CT 06520, USA; ^2^Department of Statistical Science, School of Mathematics and Computational Science, Sun Yat-sen University, Guangzhou, Guangdong, China; ^3^Zhongshan School of Medicine, Sun Yat-sen University, Guangzhou, Guangdong, China

## Abstract

Many genetic association studies used single nucleotide polymorphisms (SNPs) data to identify genetic variants for complex diseases. Although SNP-based associations are most common in genome-wide association studies (GWAS), gene-based association analysis has received increasing attention in understanding genetic etiologies for complex diseases. While both methods have been used to analyze the same data, few genome-wide association studies compare the results or observe the connection between them. We performed a comprehensive analysis of the data from the Study of Addiction: Genetics and Environment (SAGE) and compared the results from the SNP-based and gene-based analyses. Our results suggest that the gene-based method complements the individual SNP-based analysis, and conceptually they are closely related. In terms of gene findings, our results validate many genes that were either reported from the analysis of the same dataset or based on animal studies for substance dependence.

## 1. Introduction

Genome-wide association studies (GWAS) have become a powerful tool in the identification of susceptible loci for numerous diseases [1]. A typical strategy in GWAS is to analyze single nucleotide polymorphisms (SNPs) individually and select the top SNPs by setting a stringent threshold for the *P* value. Then the top SNPs were mapped into functional regions such as a gene or pathway to facilitate further investigation of the corresponding gene and disease. Based on SNP-based association analysis, many genetic variants underlying complex diseases or traits were detected [2, 3]. Due to the large number of SNPs with each of which entails an association test, it is essential to control the type I error or false discovery rate [4]. A predefined *P* value <5 × 10^−8^ is usually used as the threshold to declare a genome-wide significance SNP, which also limits the discoveries of the genes that are important to the disease. Also importantly, susceptible SNPs generally explain a small fraction of the risk—a phenomenon commonly referred to as the “missing heritability” [5, 6]. To alleviate this problem, alternative methods have emerged to complement the simple SNP-based methods. Among those methods, gene-based analysis [7–9], which jointly analyzes the SNPs within genes, is a promising solution to improve the power of GWAS. Compared with the SNP-based approach, gene-based association analysis has certain advantages. First, gene is a unit of heredity and function, and hence the gene-based association approaches can provide direct insights into the heredity and functional mechanisms of complex traits [10]. Second, from the statistical perspective, the gene-based association approaches reduce the number of association tests in the order of millions to about 20,000 gene-based tests, which dramatically reduces the chance of false discovery. In addition, the gene-based methods are not affected by the heterogeneity of a single locus. Hence, the results are highly consistent across populations [11], which enhances the likelihood of replication. 

Gene-based methods have been successfully applied to GWAS of complex diseases, including Crohn's disease [7], type 1 diabetes [12], and melanoma [8]. Despite the above-noted features of the gene-based association approach, there are few comparisons of genetic association analyses between SNP and gene-based methods. Here, we compare and relate these two approaches using the data from the Study of Addition: Genetics and Environment (SAGE) [13].

Recent studies show that there are many candidate genes associated with substance dependence. For example, GABRA2, CHRM2, ADH4, PKNOX2, GABRG3, TAS2R16, SNCA, OPRK1, and PDYN are well studied for alcohol addiction and have been replicated in many samples [13–28]. However, other candidate genes, such as KIAA0040, ALDH1A1, DKK2, and MANBA [25, 27, 29, 30], remain illusive. For addiction to nicotine, CHRNA5, CHRNA3, CHRNB4, and CSMD1 have been replicated in many studies [31–39].

Based on the analysis of the SAGE data, we report a number of susceptible loci at the SNP and/or gene levels, which validate many susceptibility loci that have been reported to be associated with substance dependence [13, 14, 25, 27, 29, 37, 38, 40–44]. Meanwhile, both SNP- and gene-based analyses reveal three novel risk genes: NCK2, DSG3, and PUSL1. 

## 2. Materials and Methods

### 2.1. Dataset and Study Design

The dataset included 4,121 subjects in SAGE with six categories of substance dependence data: alcohol, cocaine, marijuana, nicotine, opiates, and other dependencies on drugs. The data were downloaded from dbGaP (study accession phs000092.v1.p1) [13]. SAGE [13] is a large case-control study which aims to detect susceptible genetic variants for addition. The subjects were recruited from eight study sites in seven states and the District of Columbia in the United States. All subjects' life time dependencies on these six dependencies are diagnosed by using the Diagnostic and Statistical Manual of Mental Disorders, Fourth Edition (DSM-IV). All samples were genotyped on ILLUMINA Human 1 M platform at the Center for Inherited Disease Research in Johns Hopkins University. In this paper, we strictly followed the quality control/quality assurance as we did in our previous analysis [14]. Genome-wide SNP data were filtered by setting thresholds: MAF > 5% and call  rate > 90%. In addition, 60 duplicate genotype samples and 9 individuals with ethnic backgrounds other than African origin or European origin were excluded in our analysis. Finally, 3,627 unrelated samples with 859,185 autosomal SNPs passed the quality control procedures. To avoid population stratification, the dataset was stratified into four subsamples: 1,393 white women, 1,131 white men, 568 black women, and 535 black men. To capture most of the gene coding and regulatory variants, SNPs are considered being mapped to a gene if their physical locations are within 20 kilobases (kb) 5′ upstream and 10 kilobases (kb) 3′ downstream of gene coding regions [26]. In addition, SNPs are also assigned to a gene if they are in strong LD (*r* ^2^ > 0.9) with the initially assigned SNPs within the gene [10]. Together, around 533,639 SNPs were assigned to 18,699 protein coding genes (28.6 ± 47.7 (mean ± SD) SNPs per gene).

Following the conventional standards, we used 5.0*E* − 8 and 2.5*E* − 6 as the genome-wide significant thresholds for SNP-based and gene-based methods, respectively [4]. To increase the power of detecting potentially important SNPs that do not meet the stringent thresholds, we also considered relaxed thresholds. Specifically, SNPs with *P* < 1.0*E* − 5 and genes *P* < 5.0*E* − 4 were considered further. These *P* values are referred to as relaxed significance thresholds below. The selected SNPs were then mapped into the corresponding genes by the mapping rule proposed above.

### 2.2. Genetic Association Test at SNP and Gene Levels

We took several steps in testing the associations between genetic variants (SNP or gene) and substance dependenice. First, the *P* value of each SNP was evaluated by the logistic regression, and then the correlation coefficients (*r* ^2^) of all SNP pairs were calculated. The computation was performed in PLINK software (version 1.07) [45]. In the second step, we implemented the gene-based analysis in the open-source tool: Knowledge-Based Mining System for Genome-Wide Genetic Studies (KGG, version 2.0) [46] based on the association test results and LD files obtained from PLINK. Simes procedure (GATES) was employed in the gene-based association test [7]. Specifically, assume that *m* SNPs are assigned to a gene; an association test such as through the traditional logistic regression or linear regression is used to examine the association between the phenotype and each single SNP. This step yields *mP* values for *m* SNPs. GATES combines the available *mP* values within a gene by using a modified Simes test to give a gene-based *P* value. The summary *P* value is defined as

(1) $$\begin{array}{c} P_{G}=\mathrm{Min}\left(\frac{m_{e}p_{\left(j\right)}}{m_{e(j)}}\right), \end{array}$$

where *p*_(*j*)_ is the *j*th smallest *P* value among the *m* SNPs; *m*_*e*_ is the effective number of independent *P* values among *m* SNPs within the gene, and *m*_*e*(*j*)_ is the effective number of independent *P* values among the top *j* SNPs. The effective number of independent *P* values was derived by accounting for the LD structure among the specified SNPs; we refer to [7] on the calculation.

In order to compare the performance of the SNP-based and gene-based methods, in the SNP-based method, we selected those SNPs whose *P* values were less than 1.0*E* − 5 and then mapped them into the corresponding genes. This allows us to compare the susceptible genes identified by both methods discussed above.

## 3. Results

### 3.1. Detecting Susceptibility Loci at the Relaxed Significance Level

Table 1 summarizes the susceptible genes identified by the SNP-based association test and gene-based association test at the relaxed significance level. In total, 207 genes passed the relaxed gene-based threshold, whereas only 64 genes with SNPs passed the relaxed SNP-based threshold.

Next, we performed a literature search on the genetic regions which contain the identified genes and filtered the susceptible genetic regions which have been reported to associate with substance dependence for further investigation. In Table 2, we listed the filtered genes, their associated substance dependence type, the *P*  values for the gene-based method, the minimal *P*  value of SNPs within a gene, and their literature references and reported substance dependence. 

**Figure 1:**
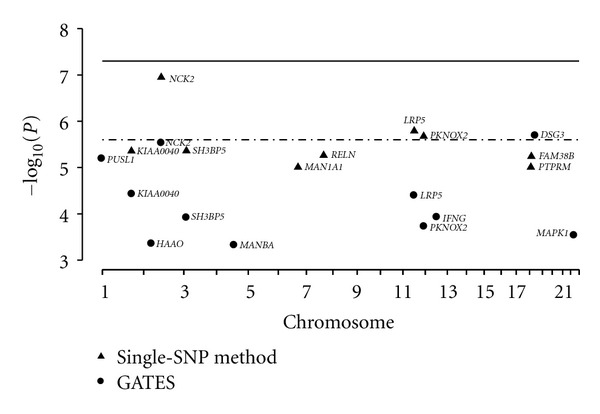
Comparison of candidate genes associated with substance dependence by the SNP- and gene-based analyses. A triangle represents the –log10 transformed *P* value of the marked gene from the gene-based analysis, and a dot represents the −log⁡10 transformed the minimal *P* value of the SNPs within the marked gene. The solid and dashed ones are the genome-wide thresholds of SNP- and gene-based significance, respectively.

In Figure 1, we plot the filtered genes obtained from the SNP-based and gene-based analyses by the position on the chromosomes against their log-transformed *P* values, −log⁡_10_⁡(*P*). Each point for the SNP-based analysis in Figure 1 corresponds to the smallest SNP-based *P* value within the gene.

Overall, five genes, NCK2 (opiates dependence in black men), SH3BP5 (cocaine dependence in white men), LRP5 (opiates dependence in white men), KIAA0040 (alcohol dependence in white women), and PKNOX2 (alcohol dependence in white women), were identified by both the SNP-based and gene-based methods as meeting either of the relaxed significance levels for a specific dependence and within a gender-racial group. Four genes, MAPK1 (marijuana dependence in black women), MANBA (alcohol dependence in white men), HAAO (cocaine dependence in white women), and IFNG (opiates dependence in white women), met the threshold by the gene-based method only. We found that the significant signal of gene MAPK1 was mainly driven by SNPs: rs7290469 (*P* = 3.25*E* − 5), rs9610271 (*P* = 4.19*E* − 5), rs9610417 (*P* = 5.38*E* − 5), and rs2876981 (*P* = 7.51*E* − 5). The *P* values for these SNPs are slightly greater than the relaxed SNP-based threshold (*P* < 1.0*E* − 5), and hence the SNP-based method failed to detect them. 

Furthermore, four other genes, FAM38B (cocaine dependence in black women), PTPRM (marijuana dependence in black women), CSMD1 (nicotine dependence in black women), and RELN (cocaine dependence in white men), contain at least one SNP that met the SNP-based relaxed threshold of significance. The gene-based *P* values for FAM38B, PTPRM, and RELN are 9.27*E* − 4, 2.21*E* − 3, and 8.53*E* − 4, respectively, which are greater than yet at the same order as the relaxed threshold (*P*  value <5.0*E* − 4). For CSMD1, 1,934 SNPs were mapped into it. Its signal was mainly determined by only five SNPs: rs2624087 (*P*  value = 8.50*E* − 6), rs4875371 (*P*  value = 4.0*E* − 4), rs2623607 (*P*  value = 6.89*E* − 4), rs10503267 (*P*  value = 7.22*E* − 4), and rs4875372 (*P*  value = 8.18*E* − 4). Because there were only 5.3% of the SNPs (103 SNPs) with *P*  value less than 0.05, the overall association from the gene became less significant.

### 3.2. Genome-Wide Significant Loci

Since none of the SNPs attained the genome-wide significance for any dependence by the SNP-based method, in this section we will only focus on the results from the gene-based method.

Table 3 presents the genes with gene-based *P* value  <1.0*E* − 5. This method identified one genome-wide significant gene, DSG3 (*P* value = 1.99*E* − 6) for nicotine dependence in white men. The *P* value of gene NCK2: 2.70*E* − 6 is very close to the genome-wide significant threshold, which provided very strong evidence for the association of opiates in black men. As shown in Table 3, both NCK2 and DSG3 contained SNPs with strong signals; they are rs2377339 (*P* value = 1.09*E* − 7) for NCK2 gene and rs6701037 (*P* value = 1.20*E* − 7) and rs1057302 (*P* value = 3.93*E* − 7) for DSG3 gene. However, none of these SNPs reached genome-wide significance.

## 4. Discussion

In this paper, we thoroughly analyzed the SAGE data from the SNP-based and gene-based methods, and compared the results obtained from these two methods. Specifically, for each sex-racial group, we performed association analysis for the six categories of substance dependence separately. The gene-based method appears to be more powerful in detecting susceptibility loci. 

Most of the genes identified in our study are supported by various reports in the literature related to the genetics of substance dependence [47, 48]. Based on some of the genes that we identified, here common genetic variants among different substance dependencies may exist [49].

Overall, we did not detect any genome-wide significant SNP when using the SNPs-based method. However, one gene, DSG3,is genome-wide significantly (*P* = 2.70*E* − 6) associated with nicotine dependence in the white men, according to the gene-based method. Another gene, NCK2, is nearly genome-wide significant (*P* = 2.7*E* − 6) in its association with substance dependence. 

The SNP-based method and gene-based method are closely related. In fact, the SNP-based method can be viewed as a gene-based method using the extreme function, namely, the minimal *P* value of the SNPs within a gene, whereas the typical gene-based method uses a weighted approach. The advantages and limitations of these two approaches are similar to those between the extreme function and a weighted average.

We should point out that both the SNP-based and gene-based methods have their own advantages and disadvantages. The SNP-based method has its unique strength in identifying genes with only a small number of significant SNPs. However, since the SNP-based method focuses on a single SNP at a time, it is less powerful to detect a gene whose SNPs have weak marginal effects, but a strong joint effect. In our analysis, 207 genes passed the relaxed gene-based threshold, whereas only 64 genes passed the relaxed SNP-based threshold. 

 Both the SNP-based and gene-based methods can be conducted conveniently in commonly available software, such as PLINK [45] for the SNP-based method and KGG [46] for the gene-based method. For the SNP-based analysis, PLINK is the most convenient platform. For the SAGE GWAS data, it took about 25 minutes to do a genome-wide SNP scan on a regular desktop computer (Intel Core 2, 4 GB Memory). In our gene-based analysis, we used the SNP-based association results and the linkage disequilibrium (LD) files from PLINK as the input to the KGG software. After this preparation, it took about 30 minutes to perform the gene-based association scan with the same desktop as mentioned above.

## Figures and Tables

**Table 1:** Summary statistics for susceptibility loci identified by gene-based method and SNP-based method.

	Alcohol		Cocaine		Marijuana		Nicotine		Opiates		Other	
	G	S	G	S	G	S	G	S	G	S	G	S
Black men	4	3	4	1	6	2	5	2	8	2	9	5
Black women	4	3	8	5	9	3	7	3	3	1	6	3
White men	16	3	9	2	10	3	4	1	11	3	3	1
White women	20	5	12	2	10	2	11	1	4	5	24	3

G refers to gene-based method. S refers to SNP-based method.

**Table 2:** Summary of the candidate genes identified by the gene-based and SNP-based methods.

Chr	Gene	Source	*P* value (gene-based)^a^	Min *P* value (SNP-based)^b^	Detected SD^c^	Reported SD	Reference
1	KIAA0040	White women	3.75*E* − 05	2.60*E* − 06	Alcohol	Alcohol	[27, 44]
2	HAAO	White women	4.40*E* − 04	3.02*E* − 05	Cocaine	Alcohol	[41]
2	NCK2	Black men	2.70*E* − 06	1.10*E* − 07	Opiates	NA	NA
3	SH3BP5	White men	1.20*E* − 04	4.24*E* − 06	Cocaine	Alcohol	[13]
4	MANBA	White men	4.63*E* − 04	3.47*E* − 05	Alcohol	Alcohol	[29]
7	RELN	White men	8.53*E* − 04	5.32*E* − 06	Cocaine	Smoking	[37]
8	CSMD1	Black women	1.23*E* − 02	8.50*E* − 06	Nicotine	Smoking	[37, 38]
11	LRP5	White men	4.01*E* − 05	1.58*E* − 06	Opiates	Smoking	[42]
11	PKNOX2	White women	1.84*E* − 04	2.20*E* − 06	Alcohol	Alcohol	[13, 27, 41]
12	IFNG	White women	1.16*E* − 04	1.57*E* − 05	Opiates	Smoking	[37]
18	FAM38B	Black women	9.24*E* − 04	5.61*E* − 06	Cocaine	Smoking	[40]
18	PTPRM	Black women	2.21*E* − 3	9.50*E* − 06	Marijuana	Alcohol	[43]
22	MAPK1	Black women	2.79*E* − 04	3.52*E* − 05	Marijuana	Alcohol	[25]

^a^ *P* value (gene-based): the *P* value obtained by the gene-based association test;

^b^min *P* value (SNP-based): the minimal *P* value of the SNPs within the corresponding gene;

^c^SD: substance dependence.

**Table 3:** Summary of genome-wide significant genes at the gene level (*P*  value < 1.0*E* − 5) and their top SNPs with *P*  value < 1.0*E* − 3.

Population	Substance dependence	Gene	Gene's *P* value	Top SNPs	SNP's *P* value
Black men	Opiates	NCK2	2.70*E* − 06	rs2377339	1.10*E* − 07
				rs7589342	1.45*E* − 04
				rs12995333	1.89*E* − 04
				rs12053259	2.31*E* − 04
				rs6747023	3.90*E* − 04
				rs879900	7.72*E* − 04

White men	Nicotine	DSG3	1.99*E* − 06	rs6701037	1.20*E* − 07
				rs1057302	3.93*E* − 07
				rs6425323	2.94*E* − 04
				rs1057239	3.35*E* − 04
